# Comparison of maxillary arch expansion using clear aligners with and without composite attachments - A finite element study

**DOI:** 10.6026/973206300223673

**Published:** 2026-06-30

**Authors:** Sanitha Dhan, Vinaya S. Pai, Mrunmayee Gholap, P. Siri Krishna, Manjunath Hegde

**Affiliations:** 1 Department of Orthodontics and Dentofacial Orthopedics, Bangalore Institute of Dental Sciences, Bengaluru, Karnataka, India

**Keywords:** Clear aligners, composite attachments, aligner thickness, maxillary expansion, finite element analysis (FEA), orthodontic aligner biomechanics

## Abstract

The influence of composite attachment geometry, placement and aligner thickness on maxillary expansion in clear aligner therapy remains inadequately understood. Therefore, it is of interest to evaluated tooth displacement and periodontal ligament stress using different attachment designs (ellipsoidal and rectangular), placements (buccal and lingual) and thermoplastic aligner thicknesses (0.5 mm and 0.75 mm). A three-dimensional finite element model of the maxillary arch was analyzed using ANSYS R18.1 to simulate expansion mechanics. Maximum displacement of the canine and first premolar was observed with a 0.75 mm aligner without attachments, while the second premolar and molar demonstrated greater expansion and higher stress concentrations with rectangular buccal attachments and 0.75 mm aligners. The null hypothesis was rejected, indicating that attachment design, placement and aligner thickness significantly affect maxillary expansion biomechanics.

## Background:

Aesthetic dentistry has grown significantly in recent years, making orthodontics an essential component of achieving functional occlusion and improved appearance. Clear Aligner Treatment (CAT), introduced commercially in the late 1990s, has become a major orthodontic modality. Its foundations were laid by Kesling [1] in 1946, who demonstrated that sequential tooth movement could be achieved using rubber-based positioners. CAT is now widely preferred for its comfort, reduced pain, better periodontal outcomes and lower risks of apical resorption [2, 3], dental trauma and microbial accumulation [4]. Its removability and transparency further enhance patient acceptance, though its effectiveness compared to fixed appliances remains debated. Systematic reviews [5] show CAT is effective for leveling, aligning, anterior intrusion, posterior buccolingual inclination and upper molar bodily movement, but less predictable for rotations and anterior labiolingual inclination. The use of auxiliaries-interproximal reduction, elastics, attachments has expanded CAT's capabilities. Aligner systems have evolved from first-generation aligner-only approaches [6] to second-generation systems incorporating attachments and elastics and third-generation systems in which software automatically places attachments and torque-inducing indentations. Composite attachments, bonded to tooth surfaces, enhance aligner retention and force transmission. Various attachment designs have been evaluated for different tooth movements [7]. Studies using finite element analysis (FEA) report that attachments can facilitate bodily movement [8] and that attachment shape and position influence efficiency [1, 10], though some findings show minimal impact on tipping forces during space closure [11]. Aligner thickness also affects treatment accuracy. It influences the magnitude and duration of orthodontic forces [12] and should be selected according to the desired movement. In aligner therapy, force is generated through a programmed mismatch between the aligner and tooth position, creating a force system [13] that drives biological tooth movement similar to conventional orthodontics. Despite the importance of attachments and thickness, the biomechanics of attachment designs for maxillary arch expansion remain insufficiently studied. The finite element method is widely used in orthodontic biomechanics [14, 15] as it accurately simulates stress distribution and tooth displacement [16]. Therefore, it is of interest to evaluate linear displacement and stress distribution on canines, premolars and molars during maxillary expansion using different attachment designs and aligner thicknesses.

## Methodology:

In this study, an existing CAD model of maxillary arch and upper teeth which was constructed from cone beam computed tomography of a patient with skeletal Class I jaw bases with well aligned teeth having normal tooth structure and shape was used (Figure 1-5 - see PDF). It was converted to STL file using mimics software.

## Maxillary Model:

The STL file was imported to HyperMesh for refining and connecting the mesh. Similarly, an occlusally bevelled rectangular and ellipsoidal composite attachment was constructed (Figure 6 & Figure 7 - see PDF). Aligners were created using HyperMesh over the teeth structure. The simulations and analysis were done through Ansys R18.1FE.

## Materials used in the study:

[1] Five Computer Aided Design (CAD) models of maxillary arch.

[2] Thermoplastic aligner model of 0.5mm and 0.75mm thickness made using HyperMesh.

[3] PDL 0.2 mm mesh size [17]

[4] Rectangular composite attachment [16] of 4x2mm with an occlusal bevel of 0.25x2x1.25mm

[5] Ellipsoidal composite attachment [16] of 4x2mm with an occlusal bevel of 0.5x2x1mm

## Software used for the procedure:

[1] Ansys R18.1 FE software for analysis mimics for conversion to STL file

[2] HypersMesh V11 for refining and creating connected mesh

## Method of collection of data:

The cone beam computed tomography of a patient with well aligned teeth having normal tooth structure and shape was taken. The image was converted into STL (Stereolithography-a popular 3D printing technology, often referred to as Standard Triangle Language or Standard Tessellation Language) software to generate the 3D geometric model. The model included the maxilla, with all upper teeth, periodontal ligament, the alveolar bone along with thermoplastic aligner models and models of composite attachments. These geometric models were converted into finite element models with the help of HyperMesh software. The elements were connected at specific locations called nodal points or nodes. The boundary conditions and loading configurations were numerically defined as displacement and forces, respectively in boundary nodes. Each element was assigned one or more parameters that defined its material behaviour (Table 1) [9, 11].

## Attachment position in the model:

Five finite element models of maxillary arch were constructed. The first model (M1) had a buccally placed rectangular shaped composite attachment [16] which bevelled gingivally on the canine, first and second premolars and first molar. The second model (M2) was with a lingually placed rectangular shaped composite attachment bevelled gingivallyon the canine, first and second premolar and first molar. The third model (M3) had a buccally placed ellipsoidal shaped composite attachment [16] bevelled gingivallyon the canine, first and second premolar and first molar. The fourth model (M4) had a lingually placed ellipsoidal shaped composite attachment that bevelled gingivallyon the canine, first and second premolar and first molar. The composite attachments were constructed and placed in the centre of the clinical crown, parallel to the occlusal plane. The fifth model (M5) was without any attachments. The expansion in all models of maxillary arch was evaluated using thermoplastic aligners both 0.5mm thickness (A1) and 0.75mm thickness (A2). The movement of 0.25 mm expansion was pre-programmed in the aligner reproducing a real clinical setting. Factors like tension-compression stress patterns in the PDL and the tooth displacement were analysed (Figure 8-12 - see PDF).

The greatest expansion occurred with 0.75 mm aligners without attachments, showing an increase from baseline:

[1] Canine width: 30.975 mm → 32.26182 mm

[2] Molar width: 57.0 mm → 58.20269 mm

## DMX (Maximum deflection):

Indicates the greatest displacement or deformation experienced by the structure under the applied load. Help determine the structural integrity and potential for excessive deformation or failure. Units are typically meters (m) or millimeters (mm).

## SMN (Minimum stress):

Represents the lowest stress level within the structure, which may be negative (tension).Provides insights into the tensile stress distribution and potential areas of minimum stress concentration.

## SMX (Maximum stress):

Indicates the highest stress level within the structure. Help identify potential points of stress concentration or failure. Units are typically in Pascals (Pa) or Megapascals (MPa). In contrast, the 0.5 mm aligners demonstrated more diffused and lower magnitude displacement, indicative of diminished control over targeted expansion forces.

## Individual tooth displacement analysis:

The comparative analysis of individual tooth displacement was conducted across various configurations of aligner thickness, attachment design and placement, with the following observations: 

## Canine: 

The maximum displacement was observed with 0.75 mm aligners without attachments (0.138068 mm). The least displacement occurred with 0.5 mm aligners and rectangular buccal attachments (0.116733 mm).

## Molar: 

The maximum displacement was found with 0.75 mm aligners and rectangular lingual attachments (0.129117 mm). In contrast, the least displacement was observed with 0.5 mm aligners and rectangular lingual attachments (0.111454 mm). These findings highlight that aligner thickness plays a more decisive role in amplifying displacement, while attachment design and position contribute to modulating the direction and control of movement. These findings confirm that all configurations were above the safe stress thresholds for the PDL, supporting the biological viability of the simulated tooth movements. Hence, the null hypothesis of the study is disapproved as there is a relative difference in the amount of expansion using Buccal and Lingual attachments of different shapes/designs or by varying aligner thickness.

## Results:

Finite Element Analysis (FEA) was conducted to evaluate the displacement pattern and stress distribution in the maxillary arch during transverse expansion using clear aligners of two thicknesses (0.5 mm and 0.75 mm), (Table 2) with varying composite attachment designs (rectangular and ellipsoidal) and placements (buccal or lingual). A total of ten simulation models were analysed using ANSYS R18.1 software, based on a refined CBCT-derived CAD model of a maxilla with Class I skeletal relationship. The CAD model included canine, premolars and molars individually segmented to extract tooth-specific displacement values. The movement of 0.25 mm expansion was simulated using the aligner reproducing a real clinical setting. The simulation incorporated detailed mesh refinements (PDL: 0.2 mm mesh) and variable aligner thicknesses enabling accurate representation of displacement gradients. A comparative analysis of individual tooth displacement across various aligner thicknesses and attachment configurations revealed distinct patterns is described in Table 3 (how far each tooth moved from its original position). For Canines, the maximum displacement was observed with 0.75mm aligners and no attachments (0.138068mm), while the minimum displacement occurred with 0.5mm aligners and rectangular buccal attachments (0.116733mm) according to Table 3. Crown-root displacement analysis was performed in this study to distinguish between tipping and bodily tooth movements during maxillary arch expansion. By assessing the differential movement of the crown relative to the root, it is possible to determine the nature of tooth displacement under orthodontic forces. Tipping is characterized by greater crown movement with minimal root translation, whereas bodily movement involves uniform displacement of both crown and root, which is biomechanically more desirable. Evaluating crown-root displacement thus provides critical insights into the effectiveness and efficiency of different attachment designs and aligner thicknesses in achieving controlled, physiological tooth movement. This analysis supports clinical decision-making by helping identify configurations that minimize adverse effects like root resorption or periodontal stress (Table 4 and Table 5). Molar has more possibility of bodily movement for 0.5 mm aligner thickness with rectangular lingual attachment since the least difference between the crown and root is observed here. Maximum displacement is observed with 0.75 aligner thickness with no attachments and lingual attachment respectively (Table 5). While individual tooth displacement provides critical insights into localized biomechanical responses, understanding the cumulative effect of these movements is essential for assessing overall treatment efficiency-particularly in cases of transverse maxillary expansion (Table 6). The displacement of each tooth contributes to the net arch widening, with canines, premolars and molars functioning as integrated units. The coordinated outward movement of these teeth under varying aligner and attachment configurations determines the degree of transverse expansion achieved across the dental arch. Therefore, analysing displacement as a segmental pattern enables the identification of movement trends, mechanical synergies and regions of differential resistance, offering a more holistic view of aligner-driven expansion dynamics. The lowest overall expansion was observed with 0.5 mm aligners and no attachments, with the inter-molar width increasing only to57.11242 mm. Buccally placed ellipsoidal attachments yielded superior expansion compared to lingual placements, confirming their efficiency in directing forces outward (Table 7). Among attachment types, ellipsoidal buccal attachments with 0.75 mm aligners provided near maximal expansion (molar width: 58.07364 mm), with improved balance between control and efficacy. When comparing aligner thickness, the 0.75 mm aligners consistently produced greater displacement across all attachment configurations, suggesting enhanced force delivery and transmission.

## Discussion:

Kesling's introduction of sequential tooth positioners marked a major shift toward customizable, removable orthodontic systems and established the biomechanical principles that underpin modern clear aligner therapy (CAT) [1]. With advancements in materials and digital planning, CAT has become an effective aesthetic alternative to fixed appliances, aided significantly by composite attachments that enhance force systems and improve the predictability of complex movements such as expansion, intrusion and rotation [6, 17]. Maxillary expansion remains an essential approach for correcting transverse discrepancies and recent studies suggest that clear aligners, when combined with optimized attachments and controlled staging, can achieve clinically meaningful transverse changes. Rossini *et al.* [18] showed effective posterior expansion with Invisalign, while Lombardo *et al.* [19] reported that outcome predictability depends on factors such as aligner thickness, material properties and the use of auxiliaries. Collectively, these findings support aligners as a viable option for mild to moderate expansion. In this study, Finite Element Analysis (FEA) was used to assess displacement resulting from different aligner thicknesses (0.5 mm, 0.75 mm), attachment shapes (rectangular, ellipsoidal) and positions (buccal, lingual) during maxillary expansion. A simulated 0.25 mm programmed expansion enabled evaluation of tooth, PDL and structural responses under each configuration. The FEA showed progressive increases in transverse displacement from canines to molars, reflecting differential force distribution across the arch. Canine-canine width increased from 30.975 mm to 32.261 mm and molar-molar width from 57.0 mm to 58.202 mm, consistent with Rossini et al. [18], who found greater posterior expansion due to reduced molar resistance. Molars displayed the highest displacement, particularly with 0.75 mm aligners and rectangular lingual attachments, whereas canines showed the most movement with 0.75 mm aligners without attachments. These findings align with Priyadarshini *et al.* [20], who reported increased force transmission with thicker aligners. Rectangular buccal attachments enhanced bodily movement more effectively than ellipsoidal or lingual alternatives, especially in molars. Conversely, 0.5 mm ellipsoidal attachments particularly lingual ones produced lower displacement and stress, mirroring the reduced intrusive forces described by Uhlir *et al.* [21] and Goto *et al.* [11]. The highest displacement and stress peaks occurred with 0.75 mm aligners and rectangular lingual attachments, consistent with observations by Gomez *et al.* [13] and Wu *et al.* [22] regarding the biomechanical efficiency of optimized attachments. Their findings that staged posterior expansion improves efficiency complement the present study's segmental displacement trends. Variability in biomechanical response between models supports the multifactorial nature of aligner-driven tooth movement, consistent with conclusions by Zhou & Guo [23]. The study further reinforces the value of FEA in orthodontics, supporting Lombardo *et al.* [19] in demonstrating its predictive capacity for aligner biomechanics. In summary, thicker aligners, especially 0.75 mm, combined with rectangular buccal attachments, delivered the most efficient and controlled expansion forces. Lear *et al.* in their respective studies stated that direct attachment shape and size had a significant effect on the orthodontic forces and moments generated by thermoplastic aligners during simulated expansion [24, 25]. These insights may help clinicians optimize maxillary expansion protocols by selecting attachment configurations and aligner thicknesses based on mechanical effectiveness and patient-specific anatomy.

## Limitations and scope for future studies:

While this study provides important biomechanical insights into stress distribution and displacement patterns during maxillary arch expansion using clear aligners with varying attachment designs and thicknesses, several limitations warrant consideration. The finite element model was based on a single patient's CBCT scan exhibiting a Class I skeletal pattern. As such, anatomical variability across different malocclusion types and craniofacial morphologies was not captured, potentially limiting the generalizability of the results. Future investigations should incorporate more advanced, time-dependent FEM simulations that integrate biological remodelling algorithms. Also studies should integrate biological remodelling factors and employ patient-specific geometries with time-dependent simulations to better mimic orthodontic tooth movement dynamics. Incorporating findings from recent biomechanical studies involving optimized aligner geometries and attachment positions can further support clinical strategies for improved efficacy of aligner therapy. Also, expanding the model sample to include diverse skeletal classes, multiple patient datasets and a wider array of attachment geometries and material types would enhance the robustness of the findings.

## Figures and Tables

**Table 1:** Material properties used in the finite element model

**Component**	**Young's modulus (MPa)**	**Poisson's ratio**
Teeth	1.96 x 10^4^	0.3
Periodontal ligament	3 x 10^2^	0.3
Alveolar bone	1.37 x 10^3^	0.3
Clear aligner	528	0.36
Attachments	12.5 x 10^3^	0.36

**Table 2:** Number of elements and nodes

**Models**	**Elements**	**Nodes**
0.5mm aligner	2457282	365273
0.75mm aligner	252388	372652

**Table 3:** Total linear displacement of individual teeth

**Aligner Thickness**	**Type of attachment**	**Attachment Side**	**Canine**	**Molar1**
0.5	No attachments	No attachment	0.122348	0.112422
0.5	Rectangular	Lingual	0.116742	0.111454
		Buccal	0.116733	0.111504
	Elipse	Lingual	0.122344	0.112404
		Buccal	0.122348	0.112421
0.75	Rectangular	Lingual	0.138055	0.129117
		Buccal	0.138063	0.129037
	Elipse	Lingual	0.136056	0.127647
		Buccal	0.136059	0.127636
0.75	No attachments		0.138068	0.129045

**Table 4:** Canine: Crown - root displacement comparison

**Aligner Thickness**	**Type of attachment**	**Attachment side**	**Canine Displacement(mm)**		
			**Crown**	**Root**	**Difference**
0.5	No attachments	No attachment	0.122348	0.002455	0.119893
0.5	Rectangular	Lingual	0.116742	0.003926	0.112816
		Buccal	0.116733	0.003928	0.112805
	Ellipse	Lingual	0.122344	0.002458	0.119886
		Buccal	0.122348	0.002455	0.119893
0.75	Rectangular	Lingual	0.138055	0.000319	0.137736
		Buccal	0.138063	0.000315	0.137748
	Ellipse	Lingual	0.136056	0.000707	0.135349
		Buccal	0.136059	0.000704	0.135355
0.75	No attachments		0.138068	0.000315	0.137753

**Table 5:** Molar: crown - root displacement comparison

**Aligner Thickness**	**Type of attachment**	**Attachment side**	**First Molar Displacement(mm)**		
			**Crown**	**Root**	**Difference**
0.5	No attachments	No attachment	0.112422	0.018137	0.094285
0.5	Rectangular	Lingual	0.111454	0.019442	0.092012
		Buccal	0.111504	0.019474	0.09203
	Ellipse	Lingual	0.112404	0.018152	0.094252
		Buccal	0.112421	0.018137	0.094284
0.75	Rectangular	Lingual	0.129117	0.020422	0.108695
		Buccal	0.129037	0.020378	0.108659
	Ellipse	Lingual	0.127647	0.020375	0.107272
		Buccal	0.127636	0.020361	0.107275
0.75	No attachments		0.129045	0.020377	0.108668

**Table 6:** Initial interarch distance

**Tooth Pair**	**Initial Distance (mm)**
Canine-Canine	30.975
First Molar-First Molar	57

**Table 7:** Transverse expansion across the arch

**Aligner Thickness**	**Attachment Type**	**Attachment Side**	**Canine-Canine(Initial: 30.975 mm)**	**First Molar-First Molar(Initial: 57 mm)**
0.5 mm	None	-	31.09735	57.11242
0.5 mm	Rectangular	Lingual	31.21409	57.22388
0.5 mm	Rectangular	Buccal	31.33082	57.33538
0.5 mm	Ellipsoidal	Lingual	31.45317	57.44778
0.5 mm	Ellipsoidal	Buccal	31.57552	57.56021
0.75 mm	Rectangular	Lingual	31.71357	57.68932
0.75 mm	Rectangular	Buccal	31.85163	57.81836
0.75 mm	Ellipsoidal	Lingual	31.98769	57.94601
0.75 mm	Ellipsoidal	Buccal	32.12375	58.07364
0.75 mm	None	-	32.26182	58.20269
